# Molecularly Imprinted Membranes

**DOI:** 10.3390/membranes2030440

**Published:** 2012-07-19

**Authors:** Francesco Trotta, Miriam Biasizzo, Fabrizio Caldera

**Affiliations:** Department of Chemistry, University of Torino, Via Pietro Giuria 7, 10125 Torino, Italy; Email: miriam.biasizzo@unito.it (M.B.); fabriziocaldera@libero.it (F.C.)

**Keywords:** membranes, molecular recognition, polymers, template

## Abstract

Although the roots of molecularly imprinted polymers lie in the beginning of 1930s in the past century, they have had an exponential growth only 40–50 years later by the works of Wulff and especially by Mosbach. More recently, it was also proved that molecular imprinted membranes (*i.e.*, polymer thin films) that show recognition properties at molecular level of the template molecule are used in their formation. Different procedures and potential application in separation processes and catalysis are reported. The influences of different parameters on the discrimination abilities are also discussed.

## 1. Introduction

The growing attention dedicated to molecularly imprinted polymer (MIP) technology has led to the development of a wide range of applications in various fields, including filtration, chromatography and sensor sectors.

Although the term “molecular imprinting” was first used in 1931 [1], practical interest in the technique did not really take off until 1972, when organic polymers with predestined ligand selectivity were prepared by Wulff and Sarhan [2] and Klots and Takagishi [3].

Molecular imprinting technology is an approach to design molecular recognition sites on materials imitating natural recognition features, such as antibodies and receptors. The MIP’s range of applications is wide: they are used in drugs and biological derivatives separation and purification, chemical sensors, catalysis. MIPs are less expensive, stronger, resistant to elevated temperatures and pressures, and are mostly chemically inert compared with other biological systems [4]. 

The mechanism of the imprinting is simple to understand: a template molecule is entrapped in a polymer matrix during polymerization, so that the molecular information is traced in polymeric material in the cross-linked powders and the molecule shape, and its complementary chemical functionality persist in the matrix network after the complete extraction of the template from the matrix as cavities [5]. These cavities are the recognition sites for the same template molecule or similar: these molecules bind to the polymer matrix with a very high specificity [6].

There are two kinds of interaction between template molecule and functional monomers: a covalent interaction and a non-covalent interaction. The former type as reported by Wulff [2] creates bonds, such as ester, and is very stable; the latter type (first reported by Haupt [7]) gives electrostatic interactions and hydrogen bonds; it is weaker and a large amount of functional monomer is randomly grafted in the matrix. The non-covalent technique has been preferentially used for different reasons: in the first instance, non-covalent procedures are easily conducted, then removal of the template is carried out simply by continuous extraction, and in the end a great number of functionalities can be introduced into binding sites [8]. The non-covalent interactions could also be studied by quantum mechanical calculations, for example in the frame of density functional theory (DFT), investigating the binding energies between the template molecule and the polymer/copolymer, because many studies have shown that hydrogen bonding and short-range electrostatic interactions are responsible for macroscopic effects [9,10,11].

Membranes can show molecular recognition. For instance, pre-modification (surface functionalization) of PP microfiltration membranes and of PET track-etched membranes were obtained by immersing the membranes in an aqueous solution of PEG400 monomethacrylate and benzophenone. The pristine and pre-modified PP and PET membranes were functionalized with poly(MAA-co-EGDMA). The membranes thus prepared were placed between two filter papers and the vessel was filled with the polymerization mixture, then irradiated with UV light [12].

A comparison between two different methods of membrane preparation was proposed by Park *et al.* [13], who employed a “post implanting” and an “*in situ* implanting” procedure for the synthesis of imprinted membranes. From a morphological point of view, the membrane prepared by *in situ* implanting has a more compact structure than that prepared by post implanting: there are many microvoids in the post implanted membrane and the microparticle size is smaller than that of the membrane prepared using the *in situ* procedure. The adsorption selectivity of the D-Phe-imprinted membrane prepared by post implanting is higher than that of the one prepared by *in situ* implanting.

In most studies performed on MIP membranes, the recognition sites are distributed in the bulk polymer phase, so their accessibility is limited, giving low membrane performance. Many studies have been carried out with a view to overcoming this problem, including, for example, an approach to the production of MIP membranes with an ordered porous structure using the method proposed by Widowski and co-workers [14]: the highly ordered porosity is produced by evaporating a polymer solution (prepared in a volatile solvent) under controlled humidity. The development of this technique was proposed also by Lu *et al.* [15] to gain ordered porosity from random poly(styrene-co-acrylonitrile) using THF as a solvent using the water-assisted method in the presence of template. SEM analysis showed the highly ordered and regular pore structure of the MIP membrane surface and the cross-section. Permeation experimentation results showed that the MIP membranes recognized the template molecule effectively and transported it with good efficiency. This could be attributed to the porous structures of the MIP membranes, because the ordered porous structures on the surface and in the cross section allow the accessibility of recognition sites, thus the MIP membrane showed the highest transport rate toward the template molecule.

The work of Ma and co-workers [16] described a new kind of polymer employed for the preparation of molecularly imprinted membranes, rarely reported in literature: the chitosan (CS). CS is a non-toxic, biocompatible, biodegradable, and amino-polysaccharide obtained from one of the world’s most abundant biopolymers, chitin. Its special properties make CS widely used in drug delivery and environmental protection applications. It can be used for membrane separation by ion exchange, chelation and adsorption.

Nanotechnology offers a new perspective for the production and the application of MIPs: it is possible to prepare MIPs as nanomaterials (nanofibers, nanospheres, *etc*.), but it is also possible to graft nanostructured MIPs onto conventional surfaces, *i.e.*, PVDF filters and glass fiber, to obtain a specific new material [17,18,19,20,21,22].

Thin inorganic or organic films with ion-sensitive field-effect transistors (ISFETs) and piezoelectric quartz crystals could be present in this type of material. According to Zayats *et al.* [23], a thin TiO_2_ film was obtained with sol-gel polymerization of a mixture of Ti(IV) butoxide and carboxylic acids (4-chloro-phenoxyacetic acid, 2,4-dichloro-phenoxyacetic acid, fumaric acid and maleic acid), thereby giving the imprinting. These films were assembled on the ISFET gate interface. An imprinted polymer membrane was then prepared by mixing AAm, 3-(acrylamido)phenylboronic acid, MBAA, *N*,*N*,*N*',*N*'-tetramethylethylenediamine and imprinted with nucleotides (AMP, GMP and CMP) to obtain the selective recognition.

A novel technique for the synthesis of MIMs imprinted membranes on glass-fiber membranes and Teflon filters was described by Ceolin *et al*. [24]. Polymer films were prepared on microporous glass-fiber membranes. Polymerization took place under an argon flow and using UV initiation. This study was carried out to optimize the synthesis procedure, template removal, rebinding tests and regeneration of the polymers. The *optimum* was found to be 1:4:20 (template:functional monomer:cross-linker), the conventionally described ratio in MIP-related literature [25,26,27].

MIPs are stiff materials that can be ground and powdered and are insoluble in all solvents. MIPs are not known as flexible films, however Sreenivasan [28] proposed a new approach to prepare and evaluate the corresponding properties of a molecularly-imprinted semi-interpenetrating polymer (semi-IPN) as a film. The monomers employed were 2-hydroxyethylmethacrylate and EGDMA and they were added to a solution of polyurethane (PU) in chloroform. The mechanical tests performed showed that MIP–IPN ultimate stress and strain is good, although less than that of PU.

The research aim to improve membrane flexibility continued with the work of Fan *et al.* [29], in which they performed, as a first step, synthesis of imprinted particles following a traditional procedure (employing MAA as functional monomer, glycol dimethacrylate as crosslinker). As a second step, the powdered MIP was added to a polysulfone (PSf) solution containing PEG 600 to improve flexibility and mechanical strength.

In addition to the polymerization onto filter plates in order to improve selectivity, it was suggested that nanofiber membranes could be synthesized by electrospray deposition (ESD) [18]. MIP membranes with a higher surface area can give two orders of magnitude higher flux and permselectivity compared with those prepared using conventional methods. ESD is a method used to obtain MIP membranes with a large surface area, consisting of polymeric nanofibers whose diameters range from nanometers to micrometers, by the action of an electric field imposed on a polymeric solution. With ESD, the morphology and the diameter of the electrosprayed membranes could be effectively controlled. 

A novel molecularly-imprinted hybrid membrane [30] was prepared using sodium alginate (SA) as the polymer and 3-aminopropyltriethoxysilane (APTES) as a precursor for introducing an inorganic component into the organic matrix and as a cross-linking agent to improve the mechanical strength of the SA membrane. When APTES content was low (<30 wt % in comparison to SA amount), almost no selective ability was found, due to the loose and excessively flexible structure. When the APTES content was higher than 40%, the MIP had a poorer ability to form template-polymer interactions. The addition of APTES increased the compatibility between the organic and inorganic phase via covalent interactions. 

In a new study by Zhang *et al.* [31], the selective recognition was evaluated with new protein MIM multi-walled carbon nanotubes (PMIM/MWNTs) were synthesized employing AAm as the functional monomer, MBAA as the cross-linker and bovine serum albumin (BSA), a protein concentration standard for many biochemical and immunological applications, as the template molecule. Comparing PMIM/MWNTs to the non-imprinted ones (nPMIM/MWNTs), the PMIM/MWNTs exhibited discriminatory recognition for BSA (2,6-fold increase in affinity) and selective ability adsorption capacities towards BSA than human serum albumin, pepsin, bovine blood hemoglobin and horseradish peroxidiase.

However, the first proposal of an easy technique for obtaining efficient MIMs with non-covalent bonds was made by Kobayashi and co-workers in 1995 [32] using the phase inversion technique to form membranes and subsequently used by the same research group [5] and by other authors [33,34,35,36,37,38]. The technique focuses on the casting of a copolymer solution of P(AA-co-AN) containing the template molecule on a glass plate, then making it coagulate in a non-solvent bath (*i.e.*, water) and finally washing the membrane with an opportune solvent in order to remove the template molecule and expose the corresponding cavities. The coagulation bath assumes great importance because it is able to give the membrane the right permeability and optimum performance, so the temperature of this bath was extensively studied in order to obtain the best results [39].

As far as membrane technology is concerned, one of the most common polymeric membranes used for molecular recognition is PAN and its copolymers. Tasselli *et al.* [40] published a study on the binding capacity of a PAN membrane, varying the amount and the type of the functional monomers (IA, AA, AAm), using the phase inversion technique in a polar solvent.

Kobayashi *et al*. [41] in 2008 proposed a novel approach to the synthesis of molecularly-imprinted membranes employing supercritical carbon dioxide (ScCO_2_) as an antisolvent, thereby inducing the phase separation of the polymer solution. Membrane preparation employing ScCO_2_ is similar to conventional immersion precipitation of polymers, but achieves better results. Advantages: since the ScCO_2_ dries the polymer membrane rapidly, it does not collapse and at the end there are no traces of organic solvents that could be removed from gaseous CO_2_ after decreasing the pressure. 

## 2. Advanced Polymeric Membranes: Synthesis and Applications

### 2.1. Pharmaceutical and Food Applications

Malaisamy *et al.* [38] studied MIP blend membranes made of cellulose acetate (CA) and sulfonated PSf with different compositions (100/0, 95/5, 90/10 and 85/15), employing the biomarker Rhodamine B (Rh B) as the template molecule and using the phase inversion method. They hypothesized that sulfonated PSf content influenced MIP surface hydrophobicity and eventually they observed that blend membranes with 95/5 composition had the highest binding capacity.

**Figure 1:**
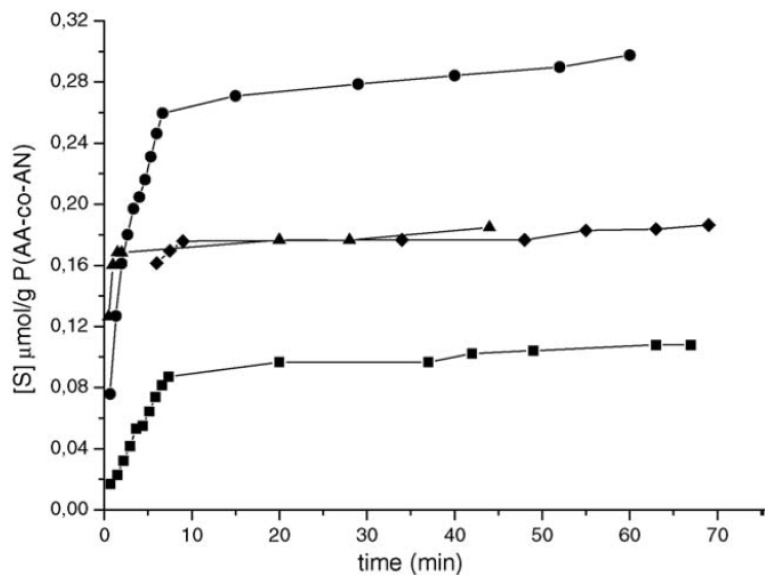
Uptaken amount of TCH and chloramphenicol in the MIM for TCH and NMIM: (■) tetracycline hydrochloride into P(AA-co-AN) NMIM; (●) TCH in the P(AA-co-AN) MIM; (▲) chloramphenicol in the P(AA-co-AN) MIM; (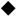) chloramphenicol in the NMIM.

Trotta *et al.* [42] suggested using P(AA-co-AN) for the production of membranes with an asymmetric pore structure, prepared using the phase inversion technique. The membranes containing the antibiotic tetracycline hydrochloride (TCH) template were prepared using the same method, but adding the required amount of the template molecule (2 wt %). Chloramphenicol, TCH analog, was used to test the selectivity of the imprinted membrane. The resulting membrane shows molecular recognition properties for the highly water-soluble TCH. About 140 μg (0.29 μmol) of TCH were retained per gram of imprinted membrane. Chloramphenicol, that has similar solubility, was less recognized (no more than 0.16 μmol/g_membrane_), as it is possible to observe in the following Figure 1.

Fan *et al.* [29] employed the chemotherapeutic agent trimethoprim (TMP) as template molecule, for imprinting particles following a traditional procedure (MAA was the functional monomer and the glycol dimethacrylate as crosslinker), then the powdered MIP was added to a polysulfone (PSf) solution containing PEG 600 to improve flexibility. The mixture obtained was cast and it was seen that when the ratios of MIP, PSf and additive PEG were 30 wt %, 20 wt % and 10 wt %, respectively, the blend membrane had selective recognition to TMP in addition to flexibility and mechanical strength.

More generally, it is possible to recognize several biomolecules in solution selectively, as studied by Silvestri *et al.* [43]: MIMs of biotechnological interest were obtained either by the coagulation or modification of NMIMs introducing imprinted nanoparticles. They observed that membranes of P(AN-co-AA) imprinted with uric acid, a marker for several diseases, such as gout, showed good recognition capacity and selectivity towards the template (the detection of uric acid was 2.4 times higher than theophylline). In addition, porous supports of EVA-dextran blends were prepared using α-amylase as a template: the selectivity of this device was 1.96 times higher than that of albumin. In some cases, the recognition properties of MAA-co-AA membranes were improved by loading imprinted cross-linked MMA-MA nanospheres. In this way, different membranes were obtained for application in the biomedical field or for various biotechnological uses, on account of their biomimetic behavior. The same author [44] subsequently suggested preparing new polymeric systems through MIP for potential application in extracorporeal blood purification. Membranes based on EVA material, produced using the phase inversion method were prepared to remove low density lipoproteins and cholesterol (LDL) from plasma employing the model compounds phosphatidylcholine (PC) and α-amylase (αA) as target molecules. In both cases, the results were positive: the selectivity of the PC-MIM was tested passing through the membrane solutions containing phosphatidylserine (PS) and phosphatidylinositol (PI), and the PC-imprinted membrane exhibited a very higher adsorption of PC in comparison to PS and PI analogs. Regarding αA selectivity and the imprinting effect, they were confirmed in a similar test by the higher uptaking of αA in respect to albumin (ALB), as is shown in Table 1.

These membranes introduced specific cavities into the polymer to bind and selectively recognize PC and α-amylase. The devices thus realized could be useful for dialysis, blood filtration, and fractioning in the biomedical field.

**Table 1:** Percentage adsorption during rebinding, selectivity, and competitive selectivity tests in respect to initial solute in the test solution of (**a**) template PC and the analogs PS and PI, (**b**) template α and the analog ALB.

TABLE (a)	% adsorbed in rebinding test		% adsorbed in competitive rebinding test	
**Molecule**	**PC-imprinted membrane**	**Control membrane**	**PC-imprinted membrane**	**Control membrane**
**PC *(template)***	78.05	6.09	68.22	4.11
**PS**	0.15	0.29	5.91	0.96
**PI**	0.10	0.16	-	-
**TABLE (b)**	**% adsorbed in rebinding test**		**% adsorbed in competitive rebinding test**	
**Molecule**	**αA-imprinted membrane**	**Control membrane**	**αA-imprinted membrane**	**Control membrane**
**αA*(template)***	43.46	12.21	39.55	9.33
**ALB**	8.30	6.12	6.71	6.89

Pegoraro *et al.* [45] focused their work on the possibility of adopting MIP based on polymeric membranes imprinted with PC for use in their research on regression of atherosclerosis. The polymer matrix was based on EVA with an ethylene molar content of 44% and three different amounts of PC template molecule (PC100 = 62.5 mg/g_membrane_; PC200 = 117.5 mg/g_membrane_), obtaining the membranes by phase inversion. Both membranes PC100 and PC200 possessed high binding capabilities (78.6% of the initial PC amount in the solution test); phospholipids similar to PI and phosphatidylethanolamine (PE) were used to test the selectivity of the membranes and both PC100 and PC200 showed selectivity for PC and not for PI and PE.

Sreenivasan [28] employed cholesterol, an important component in hormonal systems, as the template molecule, using as monomers 2-hydroxyethylmethacrylate and EGDMA, and adding a solution of polyurethane (PU) in order to obtain a semi-IPN device. Cholesterol absorption is greater in MIP–IPN (5.52 mg absorbed by 100 mg of polymer) than in control semi-IPN (0.72 mg absorbed by 100 mg of polymer) and PU (2.61 mg absorbed by 100 mg of polymer). Testosterone was chosen as a molecule with similar structure and shape to cholesterol, to evaluate MIP’s selectivity for cholesterol. These membranes imprinted with cholesterol have shown very low affinity for testosterone (0.57 mg absorbed by 100 mg of MIP–IPN; 0.62 mg absorbed by 100 mg of semi-IPN polymer; 1.87 mg absorbed by 100 mg of PU).

A MIM targeted to α-tocopherol (α-Toc) [46], a type of vitamin E, was prepared by phase inversion of polymer from a template-containing monomer, α-tocopherol methacrylate (α-TMA) and copolymerized with AN from a DMSO solution in a coagulation non-solvent water bath. The results were that the amounts of α-Toc joined to imprinted and non-imprinted membranes were 20.8 ± 0.4 and 2.2 ± 0.1 μmol/g, respectively: the imprinted membranes showed higher affinity and selectivity towards α-Toc than to the 4-chromanol analog. Evidence shows that separation was achieved with a separation factor of 15.5 for α-Toc/4-Chr using a simple filtration procedure with high flux permeation. 

The same author presented an evolution of the above study [47]: α-TMA was used as a functional monomer and copolymerized with divinylbenzene to prepare microparticles and then granulated. In this way, several hybrid MIMs (HMIP) containing the polymer powders were obtained using polymer supports such as PSf, CA and Ny. All HMIP membranes prepared using the phase inversion technique showed selective binding of α-Toc over its derivative, δ-tocopherol, exhibiting efficiencies of 0.49 for MIP powder and 0.60, 0.64, and 0.53 for PSf, Ny and CA-HMIP, respectively. Again, the same research group proposed, in 2009 [48], an MIP obtained by phase inversion targeted to α-Toc developed by polymer membrane scaffold made of PSf containing calix[4]resorcarenes and showing non-covalent host-guest interactions with the template. The amount of α-Toc bound to the imprinted and non-imprinted membranes was 41.1 ± 0.9 and 22.2 ± 1.1 μmol/g_membrane_, respectively. A separation factor of 14.5 was obtained for α-Toc relative to its analog, 2-napthol (2-Nap).

**Figure 2:**
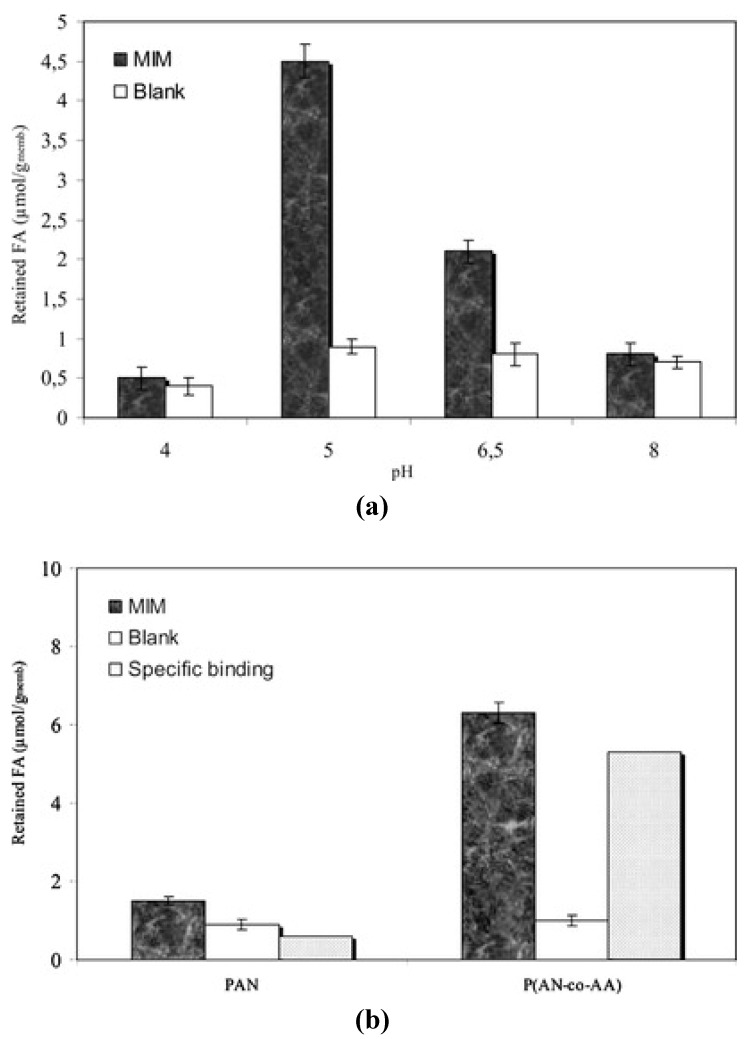
(**a**) pH effect on the binding capability of blank and MIM P(AN-co-AA); (**b**) retention of folic acid by PAN and P(AN-co-AA) prepared by solvent evaporation.

Donato *et al.* [49] suggested extracting folic acid, a constituent of the vitamin B group, from aqueous solutions, using a novel procedure based on the membrane separation process employing MIMs prepared using the phase inversion technique. The MIMs were made with poly(AN-co-Aamide) and folic acid as the template molecule. A reference sample was prepared with poly(AN). In particular, solvent evaporation made it possible to obtain poly(AN-co-Aamide) imprinted membranes, which showed a specific binding capacity of 5.3 μmol/g_membrane_. Blank membranes without the template molecule showed a low specific binding coefficient of 1.0 μmol/g_membrane_. PAN-based membranes, on the other hand, showed low folic acid retention of 1.5 μmol/g_membrane _(if prepared in the presence of the template) and 0.9 μmol/g_membrane_ (if prepared in the absence of the template). The results are reported in Figure 2.

Ma *et al.* [50] studied the release of naproxen, a nonsteroidal anti-inflammatory drug, using variables such as polymer type and concentration, solvents and casting conditions, and observed that naproxen caused a reduction in T_g_ (glass transition temperature) of the amorphous poly(lactide-co-glycolide) and poly(D,L-lactide) compared to drug loads in dry casting conditions. Release profiles for all the polymers tested followed a two-stage model: initial diffusive release, followed by zero-order release due to polymer decay.

Saljoughi *et al.* [51] synthesized asymmetric CA membranes for hemodialysis with the phase-inversion method, using PEG 400 as the additive and NMP as the solvent: increasing the PEG concentration and reducing the CA concentration resulted in increased insulin/HSA diffusion and improved the formation of macrovoids in the membrane sublayer. In addition, these conditions increased the thermal and chemical stability of the membranes. 

MIMs templated with puerarin [52] were prepared by coagulation with P(AN-co-AA). The copolymer–DMSO solution with puerarin, a recently studied plant-derived molecule having several effects on human health, was obtained at various temperatures: increasing P(AN-co-AA)’s molecular weight and decreasing the coagulation temperature (25 °C) led to an improvement in puerarin recognition by the membrane and also in the purity of puerarin, which rose from 56.51 to 98.41 wt %. 

Zhang *et al.* [53] used oleanolic acid (OA), a molecule that exhibits antitumor and antiviral properties, as the template molecule, polyamide-6 (PA6) as the membrane and PSMA to prepare PA6/PSMA-OA molecularly imprinted composite membranes by the phase inversion method in ScCO_2_. They tested different conditions to obtain the *optimum*, *i.e.*, the mass ratio between PSMA and OA (from 3:1 to 8:1), the temperature of ScCO_2_ (from 35 to 50 °C) and the pressure of ScCO_2_ (12 MPa to 17 MPa), finding that the highest adsorption rate and purity of OA were 50.41% and 96.15% respectively. 

One of the aims of MIP-related research is to increase selectivity and the extent of the gate effect (the morphology and diffusive permeability of the MIP membrane is affected by different issues that play a key role in the recognition properties of MIP membranes and it is very important to control them). These issues are polymer flexibility, density, the amount of specific binding sites and the swelling/shrinking capability in the presence of the print molecule. To optimize these factors, it could be possible to control the radical polymerization in MIP synthesis. Living radical polymerization is induced by “*iniferter*” (*ini*tiator-trans*fer* agent-*ter*minator), which acts as an initiator, retarder, terminator and other transfer reactions. The degree of polymerization and the primary structure of the synthesized polymer can be conventionally controlled by reaction time and the degree of branching by the time of ultraviolet (UV) irradiation [54]. Living polymerization was employed for the grafting of a polymer imprinted with the bronchodilator theophylline using photoactive iniferter immobilized on the cellulose dialysis membrane surface. The surface morphology of the MIP membrane varies with time of polymerization and UV irradiation. 

An approach to the ESD technique was proposed by Yoshimatsu *et al*. [19] using PET as a support for imprinted nanoparticle encapsulation. The study was performed to detect traces of propanolol in an aqueous solution containing (*R*,*S*)-propranolol hydrochloride, (*S*)-propranolol hydrochloride and (*R*)-propranolol hydrochloride. The interesting characteristic of these nanofibers is that there is no loss of particles, so the same composite nanofiber could be reused more than 10 times after regeneration, without loss of this property. The imprinted composite nanofiber membrane tested can selectively extract propranolol from solution samples.

The flavone luteolin, with antioxidant effects, was used as the template molecule employed by Zhang *et al*. [55] for the preparation of composite membranes for grafting the upper side of the Al_2_O_3_ microporous asymmetric tubular membranes. APTES is the functional monomer and TEOS as the crosslinker. Rutin (RT) was used as the competitive recognized molecule, because its molecular structure is similar to luteolin, but there is a big difference between their permeability performances through the composite membrane: the test performed on a mixture showed that the estimated separation factor of luteolin and RT was 14,12.

**Figure 3:**
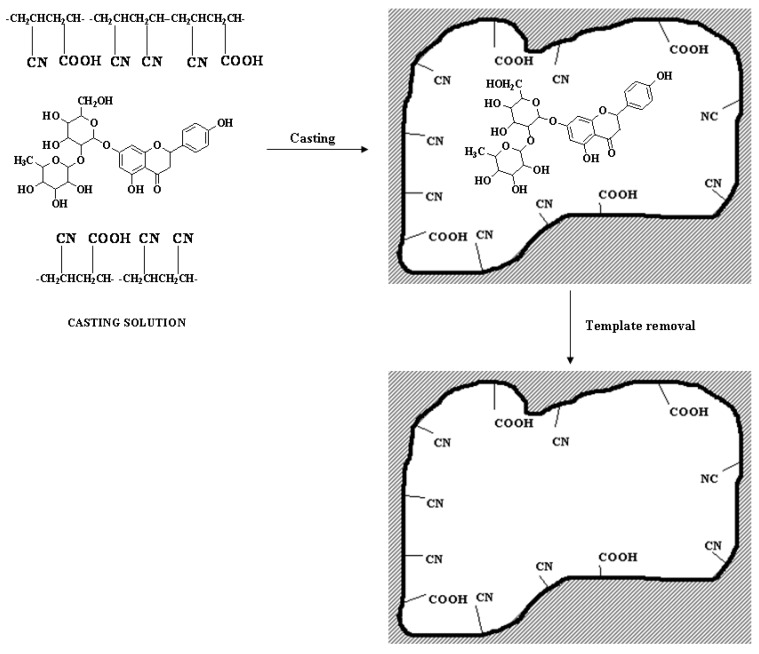
Scheme of NG-MIM preparation.

Trotta *et al.* [56] studied the retention of the flavonoid naringin (4,5,7-trihydroxyflavanone-7-rhamnoglucoside) (NG), a molecule that like limonin, hesperidin and other molecules contributes to the bitter taste of orange juice, being present in the rind of citrus fruits. The co-polymer employed was P(AA-co-AN) with 16.6 mol% of AA and was synthesized with template molar content 2%–4%. The scheme of the preparation is shown in Figure 3. Molecular imprinted membranes, as expected, were able to bind NG effectively, whereas the non-imprinted membrane did not show any retention property for NG. The greater amount of NG entrapped in the P(AA-co-AN) membrane (4%), does not lead to an increase in the retained amount of the template, presumably because most of the NG binding sites are not correctly positioned or are inaccessible.

Tasselli *et al.* [40] published a study on the binding capacity of different PAN copolymer membranes with NG. Four kinds of membrane were obtained using the phase inversion technique in a polar solvent: polyacrylonitrile (PAN), poly(acrylonitrile-co-itaconic acid) (P(AN-co-IA), poly(acrylonitrile-co-acrylic acid) (P(AN-co-AA) and poly(acrylonitrile-co-AAm) (P(AN-co-AAm) at two ratios: (3:1 and 6:1). All membranes presented good specific recognition properties, especially P(AN-co-Aamide), which showed the best overall (12.9 μmol/g_membrane_) and specific (9.0 μmol/g_membrane_) binding capacity.

NG was also the target molecule for a study by Ma *et al*. [16]: a MIM was prepared in aqueous media using CS as functional polymer, NG as template molecule, PEG as porogen and H_2_SO_4_ as crosslinking agent and the membrane was obtained by the phase-inversion technique. The MIM showed excellent performance with the mass ratio CS:NG = 15:1. The NG–CS MIM was used to separate NG from aqueous mixtures of NHD and NG and the highest permeation percentage was 11.16% for eight hours.

**Figure 4:**
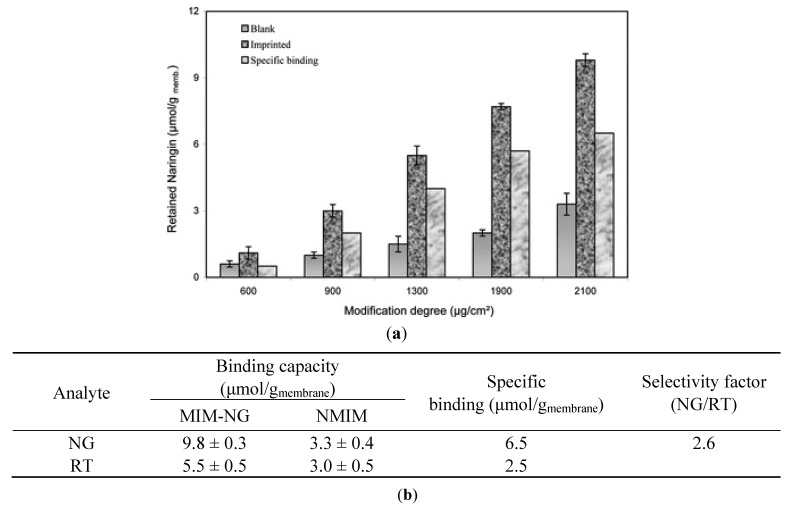
(**a**) NG retention on poly(4-VP/PVDF) NR-imprinted and its corresponding blank membrane at different modification degree; (**b**) Binding properties of NG-imprinted membrane and its corresponding blank.

NG recognition was also achieved by Donato *et al.* [57] by using surface functionalization of PVDF microfiltration membranes, conjugating the imprinting and the membrane technology. They made use of 4-VP as the functional monomer and EGDMA as the crosslinker, with the help of benzoin ethyl ether (BEE) as the photoinitiator, using varied concentrations of the 4-VP and EGDMA to obtain membranes with different modification degrees. As it is possible to observe in the Figure 4(a), the highest specific binding was found in MIMs with modification degree value about 2100 μg/cm^2^ and after the selective recognition test performed with the structural analog RT, it has been possible to assert the stronger affinity of this new kind of MIMs for the template than the analog.

**Figure 5:**
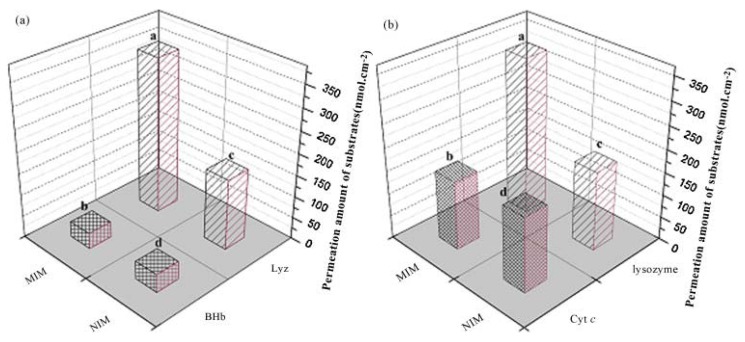
Selectivity activity of MIM and NMIM towards mixture containing lysozyme and BHb (**a**) and Cyt c (**b**).

To perform a permselective separation of lysozyme, a very important protein in human health, Chen *et al.* [58] presented a novel method to obtain a polymeric membrane based on a copolymer P(AN-co-DTCS) onto which AA and MBAA were crosslinked in the presence of lysozyme as the template molecule, by UV irradiation. The membranes so obtained were tested with a mixture of lysozyme and BHb or lysozyme and cytocrome c (Cyt c) and the results showed that MIMs have high selectivity performances towards lysozyme and not towards BHb and Cyt c, giving a selectivity factor of 2.51 for lysozyme *vs.* BHb and 2.13 for lysozyme *vs.* Cyt c, as it is possible to see in Figure 5.

Particular attention has to be focused to the sensoristic applications of MIMs.

Propofol (2,6-diisopropylphenol) is an intravenous anesthetic with a short sleep-induction period. Metabolic rates vary from one individual to another, making continuous monitoring of propofol preferable. The most suitable monitoring system for an anesthetic would be an on-line biosensor using an antibody or a molecularly imprinted polymer (MIP)-based detection system. Petcu *et al.* [59] proposed the synthesis of a propofol-recognizing polymer to obtain a detection system for a sensor application for blood solutions: they employed EGDMA as crosslinker, 1,1'-azo-bis(cyclohexanecarbonitrile) as catalyst and 4-acetoxystytrene. They polymerized the membrane over polytetrafluoroethylene (PTFE), cellulose and nylon filter membranes and tested the selectivity in blood solutions spiked with known amounts of test-molecules. The results showed it to be a good method for the recognition of propofol and the test was bias-free.

MIMs were also used to concentrate and analyze propanolol, a beta-blocker drug, in urine and blood samples, and work concerning this application was reported by Renkecz *et al.* [60]. Oxprenolol was used as an analog to test membrane selectivity. The cross-linked polymer was prepared from MAA and EGDMA. Propranolol was detected from urine samples around the minimum required performance limit and from blood samples in the typical relevant concentrations. Specificity, selectivity and repeatability tests confirmed that this new method matched the requisites for biological sample analysis.

In the field of antibiotics, Rebelo *et al.* [61] published a study on MIMs for the TMP, in which they described the preparation of new TMP MIM-based ion-selective electrodes. The polymeric sensor was synthesized with methacrylic acid 2-VP functional monomers, including the template molecule. The sensing material was dispersed in a PVC matrix and plasticized with *o*-nitrophenyl octyl ether. All sensors with MIMs revealed a linear behavior against the logarithm concentration of TMP along a wide concentration range.

A new biomimetic sensor for enrofloxacin, was prepared by Kamel *et al.* [62], who described the development of enrofloxacin MIP-based ion-selective electrodes. The sensor is produced with methacrylic acid and/or 2-VP templated with enrofloxacin. The sensing materials thus built are dispersed in a PVC matrix plasticized with o-nitrophenyl octyl ether (oNPOE). The effect of pH on the potentiometric response was investigated in acetate or Tris buffers with a pH range of between 4 and 9. The sensitivity of the sensors was stable from pH 4 to 7 and dropped above pH 7. Both MAA and MAA-VP based sensors showed good potentiometric analytical properties, able to distinguish enrofloxacin from other fluoroquinolones in sample solutions. 

With a similar synthesis procedure, Guerreiro *et al.* [63] produced new chlortetracycline ion-selective electrodes with the intention of enhancing selectivity with the improvement of analyte recognition through MIPs. The new sensor was synthesized with MAA and AA functional monomers and cross-linked by EGDMA containing the template molecule. The sensing materials were then distributed in a PVC matrix and plasticized with oNPOE. The sensors were used to analyze contaminated fish, synthetic urine and serum samples. The MAA-based MIPs had a greater affinity for the template than AA-based ones. Two different operational pH ranges could be indicated for these electrodes: 1.5 to 3 and 7 to 12 pH. 

Almeida *et al.* [64] proposed the construction of sulfadiazine and sulfamethoxazole selective electrodes based on imprinted sol-gel (ISG) material. The ISG was employed as the electroactive material on PVC membranes selective for sulfadiazine and sulfamethoxazole, prepared by the inclusion of ISG particles into the PVC matrix. The sol-gel membranes were obtained by coating a graphite support with a small amount of the viscous ISG solution. The best performance was given by ISG particles in PVC, concluding that these sensors are appropriate for real sample applications. The proposed sensors for sulfadiazine and sulfamethoxazole operated suitably under laboratory conditions, with good precision of the analysis for both sulfonamides.

### 2.2. Polymer Membranes for Chiral Recognition of Amino Acids and Nucleic Acids

In 1997, Yoshikawa *et al.* [65] presented alternative molecularly-imprinted polymeric membranes prepared from a polystyrene resin bearing D-amino acids or L-amino acids. The membrane prepared from a DLDE derivative made of D-amino acids and imprinted by Boc-D-Trp recognizes the D-isomer in preference to the corresponding L-isomer. Steric effects, interaction between the carboxyl group in the print molecule and the amino group in the tetrapeptide residues are considered important factors and electrodialysis of the racemic amino acid solution shows that permselectivity directly reflects its adsorption selectivity. In a later work [66], protected amino acids Boc-L-Glu(OBzl), Boc-L-Gln, Boc-L-Lys(4-Cl-Z), and Boc-L-Leu/H_2_O were used as the imprinting molecules and the membrane materials were prepared using the Merrifield technique. The membrane imprinted with Boc-L-Trp or Ac-L-Trp, showed selectivity to the print molecule family. On the other hand, the membrane containing tetrapeptide residues of L-amino acids and imprinted by an L-amino acid derivative, recognized the L-isomer over the D-isomer. 

A similar work was proposed on molecularly-imprinted polymeric membranes prepared from non-chiral synthetic polymer carboxylated PSf [67]. Z-D-Glu or Z-L-Glu was adopted as print molecules. Membranes imprinted by Z-D-Glu recognize the D-isomer over the corresponding L-isomer, and *vice versa*. The amino acid preferentially adsorbed by the membrane was also selectively permeated by electrodialysis.

A more recent development of the previously cited works [68] consists of a polystyrene resin containing tetrapeptide of Gly and using the D- or L-isomer of Boc-Trp as a print molecule. The membrane imprinted with the D-isomer recognized the Ac-D-Trp well and the one imprinted with the Boc-L-Trp had specific recognition for Ac-L-Trp. These two types of membrane exhibited optical resolution ability and there was adsorption selectivity in enantioselective electrodialysis.

Dzgoev *et al.* [69] successfully used MIP technology to obtain a membrane that showed enantioselectivity in order to distinguish between two enantiomers of *N*-carbobenzyloxy-L-tyrosine, employing for the synthesis of the membrane 1,1,1-tris(hydroxymethyl)propane trimethacrylate, MAA and AIBN. The same authors group prepared a Phe-imprinted membrane with two different methods: *in situ* implanting and post implanting, resulting in *in situ* D-Phe imprinted membranes thicker than the post implanting ones. The membranes prepared by the post implanting method therefore presented certain advantages: they selectively adsorbed D-Phe from a racemic solution, there are a great many macrovoids distributed in the matrix and the size of microparticles is smaller. In this way, the adsorption of D-Phe with post-implanting membranes is far higher than with *in situ* implanting membranes. 

**Figure 6:**
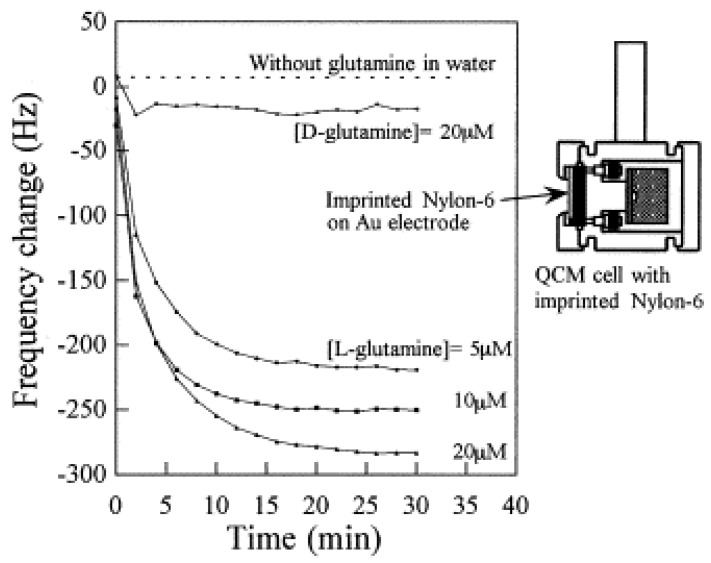
Frequency change of the L-Gln imprinted polymer-QCM sensor for L-Gln and D-Gln.

Reddy *et al.* [37] employed another kind of polymer (Ny6) to set up an L-Gln detection property both by heterogeneous batch and QCM electrode measurements. They carried out binding experiments in aqueous L-Gln, D-Gln, L-Glu and D-Glu solutions. The recognition experiments were extended to membrane filtration and quartz crystal microbalance response using the imprinted Ny6. In the batch binding experiments, the high recognition of L-Gln was confirmed by the imprinted polymer. The reduction of the frequency depends on the L-Gln concentration: the highest concentration of the L-Gln (20 μM) caused a big frequency change of the QCM. In contrast, the frequency reduction for the D-Gln solution was much lower in comparison to that detected in the L-Gln. Thus, the Nylon-6 MIM imprinted with L-Gln bound the L-Gln molecules with high affinity, as it is possible to observe in Figure 6.

Another polymeric support was employed by Wang *et al.* [39]: a modified sol-gel process, using CS and glycidoxypropyltrimethoxysilane (GPTMS), led to a dense and uniform enantioselective hybrid membrane (CS/GPTMS) with a low degree of swelling. It was imprinted with L-Phe and efficiently applied in chiral resolution of a D,L-Phe racemic mixture. The imprinted cavities gave considerable improvement in chiral resolution, reinforcing the binding ability and delaying their diffusion.

Ny6, Ny6,6 and terephthalic phenylene [70] polyamide (TPPP) were functionalized by phase inversion molecular imprinting to add L-Phe binding sites. Formic acid was used as the solvent and the solutions had 20 wt % nylon and 8 wt % L-Phe. The resulting porous membranes behaved as membrane adsorbents that separated the L/D mixture of the substrate. The imprinted Ny6 and Ny6,6 presented high selectivity to the L-form substrate with respect to the TPPP membranes, but the imprinted TPPP membranes showed higher binding capacity with 0.57 μmol/g for L-Phe. The partition coefficients of L- and D-forms by the imprinted membranes were 6.8 for Ny6, 4.2 for Ny6,6 and 1.7 for TPPP. The imprinted Ny6, Ny6,6 and TPPP membranes had separation factors of L- and D-Phe of 1.1, 1.1 and 1.2, respectively. 

**Figure 7:**
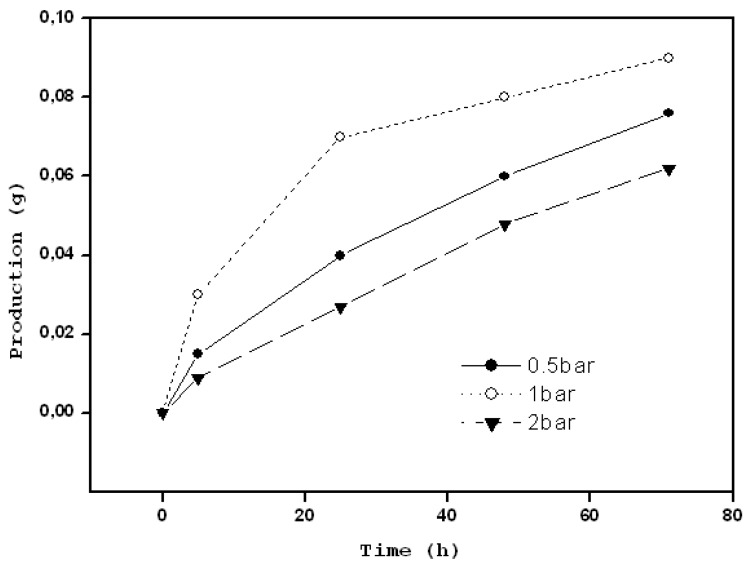
Separation of the D- and L-serine by the MIPCM from Ser racemate as a function of operating time as the operating pressure was varied from 0.5 to 2 bars.

In order to successfully separate another aminoacidic racemic mixture (D,L-Ser), Son *et al.* [71] prepared an MIP composite membrane (MIPCM) with PSf as the polymeric support and D-Ser as the target molecule. The preparation was carried out by interfacial polymerization and the MIPCM obtained gave a good optical resolution of D- and L-Ser (Figure 7). The efficiency of the separation depended on the operating pressure, for the reason that the intensifying operating pressure does not always lead to positive results: by increasing operating pressure from 2 to 3 bar, the amount of D- and L-Ser that passed through the membrane improved, but the composition of the D- and L-Ser in permeates became different, because the L-Ser seemed to compete with D-Ser to pass through the chiral space, diminishing the quantity of D-Ser in permeates and increasing that of L-Ser. Consequently, employing operating pressure under 2 bar, the authors observed that the best results were realized at 1 bar.

For the resolution of the water-soluble amino acid D,L-Phe racemic mixture molecule, Ul-Haq *et al.* [72] proposed and successfully developed enantioselective D- and L-Phe-imprinted AA/AN membranes. Recognition cavities were effectively formed in the prepared membranes, which had a nanoporous structure. The D- and L-Phe-imprinted membranes achieved rejection selectivities of 0.13 and 0.28, adsorption selectivities of 2.25 and 2.40 and permselectivities of 1.94 and 2.08 respectively. 

In 2010 [73], the same author proposed another enantioselective D-Phe imprinted P(AA-co-AN) membrane prepared by phase inversion precipitation. The membrane selectively adsorbed template enantiomer over the other enantiomer and adsorption selectivity was higher at low solute concentrations and had a rejection selectivity of 0.82–0.64 and 0.91–0.63 for the filtration of 100 and 10 ppm racemic solutions. The membranes used were nanoporous without macrovoids and enabled the optical resolution of Phe. 

The reaction of lithiated PSf with the chiral terpenoid myrtenal led to a polymeric material having a chiral performance [74]. Molecularly-imprinted membranes were prepared with the myrtenal-PSf in the presence of print molecules (D,L-Glu). The control non-imprinted membrane also showed permselectivity for racemic Glu mixtures, but as with the other works, they found that the Z-D-Glu imprinted membrane showed D-isomer adsorption and diffusivity selectivity, and the same behavior was observed for the L-isomer. 

Using a similar procedure, Hatanaka’s research group [75] synthesized novel polyureas with a chiral attribute prepared from L-lysine ethyl ester and 1,4-phenylene diisocyanate and investigated their optical resolution ability. They adopted *N*-α-protected Glu (Z-D-Glu or Z-L-Glu) as print molecules. Contrary to expectations, the polyurea control membrane showed adsorption selectivity. The D-isomer of Z-Glu imprinted membrane adsorbed, as expected, the D-isomer of Glu instead of the corresponding L-isomer and similar behavior was observed for the L-isomer. However, the Z-L-Glu imprinted membrane showed permselectivity for D-Glu, although L-Glu was incorporated into it: this inconsistency between adsorption selectivity and permselectivity was explained by the delayed transportation through the membrane of the enantiomer included in the membrane. Those MIMs showed chiral separation ability if a concentration gradient was adopted or potential difference was applied as a driving force for membrane transport.

Selective adsorption of Z-D-Glu was also proposed by Sueyoshi *et al.*, employing as polymeric support nanofibers produced by ESD prepared from CA and Z-D-Glu as the print molecule. In addition to the proven permselectivity (D-Glu was transported through the Z-D-Glu molecularly imprinted nanofiber membrane in preference to the corresponding L-Glu and vice versa), these nanofiber molecule allowed an enhancement of the flux employed to pass the test solution through the membrane about two orders of magnitude higher than the usual MIM [76]. The same authors have recently presented an evolution of the previous work [77], employing PSf aldehyde derivatized nanofiber membranes: the target molecules were the same and a study on the influence of the flux through the membrane was performed, leading to the conclusion that the nanofiber aldehydic PSf also allowed an enhanced flux about two orders of magnitude higher than the usual MIM tests.

Yoshikawa *et al.* [18] made use of MIP electrosprayed nanofiber membranes prepared from carboxylated PSf and employing Z-D-Glu or Z-L-Glu as a template molecule. The results of the study reported that Z-D-Glu MIP nanofiber membranes have a preferential recognition for the D-isomer than the corresponding L-isomer, whereas Z-L-Glu MIP has high recognition for L-isomers. 

MIM technology is also very useful for studying nucleotides. Yoshikawa *et al.* [78] used 9-ethyladenine as a print molecule and investigated the recognition and selective transport of adenosine and guanosine mixtures. The printed polymers were polystyrene resin (DIDE-resin), cellulose acetate and polysulfone. The MIMs synthesized in this way recognized/adsorbed adenosine instead to guanosine. However, guanosine was preferably permeated over adenosine, probably because of the relatively high affinity between adenosine and membrane.

According to Zayats *et al.* [23], a thin TiO_2_ film was obtained with sol-gel polymerization of a mixture of Ti(IV) butoxide and carboxylic acids (4-chloro-phenoxyacetic acid, 2,4-dichloro-phenoxyacetic acid, fumaric acid and maleic acid), thereby giving the imprinting. These films were assembled on the ISFET gate interface. An imprinted polymer membrane was then prepared by mixing AAm, 3-(acrylamido)phenylboronic acid, MBAA, *N*,*N*,*N*',*N*'-tetramethylethylenediamine and imprinted with nucleotides AMP, GMP and CMP. Selective detection by the imprinted sites was observed, which can be attributed to the complementary H-bonds between the nucleotide and the AAm units: in the case of AMP-imprinted AAm-acrylamidephenylboronic acid copolymer increased the gate-source potential change varying concentrations of AMP; in the presence of GMP and CMP the device had a low response. In the same way, the membrane imprinted with GMP and with CMP showed the same behavior in the presence of GMP and CMP respectively.

Sallacan *et al.* [79] created an AAm-acrylamidephenylboronic acid copolymer membrane with molecular recognition sites for the nucleotides AMP, GMP, CMP, and UMP, and also specific recognition sites for β-D(+)-glucose, D(+)-galactose, and β-D(−)-fructose. The membranes were built on piezoelectric Au quartz crystals or Au electrodes via electropolymerization or on the gate surface of an ISFET device by radical polymerization. The speed of the swelling process was slow, so the response times of the microgravimetric measurements were proportional to the swelling process, whereas the response time of the ISFET device was faster, but the microgravimetric and electrochemical analyses are one hundred times less sensitive than the ISFET devices.

Uracil, a molecule often employed for drug delivery, was selected by Wang *et al.* [39] as a template for preparing molecularly-imprinted membranes of poly(AN-co-MAA). This could be one application for bio-mimetic artificial components for studying RNA in biological organisms. Permeation experiments employing uracil or dimethyluracil showed that the imprinted membranes effectively recognized the template molecule.

Also Kobayashi *et al*. [41] used uracil as template molecule and prepared microporous PSMA membranes using ScCO_2_ as a nonsolvent for the phase inversion technology. Compared with water, the membrane prepared in ScCO_2_ showed regular cellular structure and no finger-like morphology. It also depends on the solvent used. They have studied the effect of DMF, DMSO and NMP, and the NMP gave a slightly isolated large pore size, whereas with DMF and DMSO the pore size was not isolated, rather it was interconnected. Uracil binding to the imprinted membrane prepared in ScCO_2_ was higher than that prepared in water: this occurs because most of the uracil dissolves in water during polymer coagulation. The results indicated that the URA-imprinted membrane prepared at 50 °C recognized and selectively bound URA than was the case at 35 °C. The resulting MIMs prepared at 35 °C and 50 °C bound URA with 9.2 ± 0.10 and 12.6 ± 0.06 μmol/g, respectively. Competitive binding studies were carried out with a solution containing URA/DMURA, URA/Thymine, and URA/Cytosine. The URA-imprinted membrane showed a high separation factor (α) of 17 for both URA/1,3-dimethylURA and URA/Thymine, and for URA/Cytosine, α = 13.

In their work, Pogorelova *et al.* [80] reported a new method for obtaining ISFET devices imprinted with specific recognition sites for NAD^+^, NADP^+^, NADH, and NADPH with a cross-linked AAm-acrylamidophenylboronic acid copolymer associated with the Al_2_O_3_ gate surface. The results demonstrated the successful imprint of the specific recognition sites for the oxidized cofactor NAD^+^ or NADP^+^ or the reduced cofactor NADH or NADPH and the assembly of functional sensing devices for the substrates. The observed selectivity existing for the oxidized pairs NAD^+^ and NADP^+^ or the reduced cofactors NADH and NADPH is remarkable: selectivity was induced by the additional single H-bonds given by the phosphate group present on the ribose unit of NADP^+^/NADPH.

### 2.3. Metal Ions

Due to the biological and environmental impact of metal ions, the development of new methods for selective separation, purification and determination of these compounds is of continuing interest.

Li *et al.* [81] reported the technology of using a Cu^2+^ template for nitrocellulose membrane-poly (vinyl alcohol)-ionic imprinting (NCM-PVA-I-I). In a condition of common cation and anion coexistence, NCM-PVA-I-I can distinguish copper with high selectivity, because the cavity in NCM-PVA-I-I is complementary to Cu^2+^. The Cu^2+^ entered the cavity and then formed an ionic association with the fluorescein anion outside the cavity by electrostatic effect.

Zhuqing *et al*. [20] reported a procedure for imprinting functional groups on sol-gel silica nanotubes for copper ion separation, using *N*-[3-(trimethoxysilyl)propyl]ethylenediamine (AAPTS) and CuSO_4_·5H_2_O to give Cu[AAPTS]_2_^2+^ complex, and TEOS was added to form silica nanotubes. The best pH range for the extraction of Cu(II) was from 5 to 7.5, thereby avoiding the precipitation of the metal hydroxide and the protonation of the amine as the chelating groups. Adsorption and ion-recognition studies were carried out with Cu(II) and Zn(II) ions, and it was observed that the Cu(II) ion-imprinted silica nanotube membranes had a high adsorption capacity for Cu(II). 

A QCM is an extremely sensitive surface sensor capable of measuring a nanogram level change in mass on the surface. QCM applications in biochemistry, environmental, food, and clinical analysis are very attractive, since this technique provides a label-less method for the direct study of biospecific interaction processes (e.g., the affinity interaction between antigen/antibody). Cai *et al.* [21] presented a study about the adsorption of metallothionein (MT) with the nanocrystalline TiO_2_ membrane on an electrode covered with a protein layer using a QCM. The crystals obtained were treated with MT solutions. It can be observed that adsorption of NIM to MT changes little for pH ranging from 7.1 to 10.3, whereas the change is obvious for NIM to MT. Actually, the washing of MIM leaves free cavities in the structure that are able to bind MT molecules better than NIM and selectively with respect to molecules of a similar structure.

A new approach was proposed for preparing a metal ion-imprinted polymer membrane through *in situ* polymerization using the Zn(II)-(2,2'-bipyridyl) complex as the template, 4-vinylpyridine (4-VP) as the monomer and PVDF membranes as the supporting material [82]. The imprinted membranes revealed higher selective adsorption and permeation for the template than the control non-imprinted membranes. Selective permeation of Zn(II) over Cu(II) was observed: the species with the fastest permeation was Zn(II)-*o*-diaminobenzene. The second fastest permeation was for Zn(II)-2,2'-bipyridil, followed by Cu(II)-2,2'-bipyridyl. Zn(II)-2,2'-bipyridyl and Cu(II)-2,2'-bipyridyl had a similar size, however, the simple small difference between Cu and Zn made the imprinted membrane selective for Zn(II) because of the size of cavities formed on the supporting membrane.

Another study on the permeability of 2,2'-dipyridyl as a solution to molecular recognition in a strong polar solvent was proposed by Wang *et al.* [83], employing porous PVDF a hollow fiber ultrafiltration membrane as a flexible and mechanically stable support. This study focused on the behavior of Ni^2+^, which plays a key role in the recognition process, and the binding target was [Nidipy]^2^^+^ complex. Various factors affect membrane permeation performance, such as ion concentration, cations and counterions and pH. Changes in pH make it possible to adjust the permeation performance of MIMs, making them suitable for use in controlled drug release applications. 

As mentioned in paragraph 1, composite membranes are very interesting for various purposes. One of the most important aims is the recognition/adsorption/selective removal of metal ions. Sodium alginate (SA) was employed in combination with polyvinyl alcohol (PVA) and PEG to obtain a porous composite membrane imprinted with Cr(NO_3_)_3_·9H_2_O for the selective adsorption of Cr(III) ions (Cr(III)-PVA/SA) [84]. The effects of different parameters were evaluated in order to reach the best condition for the employment of the composite membrane: the concentration of template Cr(III) ions was found to be best at 0.078 wt %, the pH value of the solution had to be about 6.0 and temperature increases had a proportional effect on adsorption. The adsorption ability of Cr(III)-PVA/SA for Cr(III) ions peaks at 59.9 mg/g_membrane_. Competitive adsorption studies were performed for the Cr(III)/Cd(II), Cr(III)/Cu(II) binary mixed system and the Cr(III)/Cd(II)/Cu(II) ternary mixed system: the MIP Cr(III)-PVA/SA is highly selective to Cr(III) ions due to the imprinted cavities in the adsorbent. The adsorption–desorption experiment shows that the Cr(III)-PVA/SA has an efficient reusability. 

Vatanpour *et al.* [85] synthesized imprinted and non-imprinted membranes using Ni(II) ions and diphenylthiocarbazone ligand, employing MAA, EGDMA and AIBN. The study was carried out at different pH values: at pH 5, the extraction of Ni ion was around 32%; Ni ion adsorption increased with an increase in the pH of the solution from 7 to about 8. Above a pH of 8, the sorption of Ni ions decreased. To test the selectivity of Ni(II) *versus* Co(II) ions, pH 7 was employed and the selective permeation of Ni^2+^ versus Co^2+^ was observed. After several cycles of adsorption/desorption, the adsorption capacity was preserved at around 90% of the pristine membrane.

Cross-linked CS presented lower adsorption capability because of functional groups (-NH_2_) being cross-linked. Ion imprinting for the cross-linked CS adsorbent proposed by Shawky *et al.* [86] overcame this problem. The aforesaid authors used Ag^+^ as the imprinted metal ion in membrane synthesis. Competitive removals of Ag^+^/Cu^2+^ and Ag^+^/Ni^2+^ from mixtures were also studied: the non-imprinted membranes are selective for Cu^2+^ and Ni^2+^. CS Imprinted membranes showed relative selectivity coefficients for Ag^+^/Cu^2+^ and Ag^+^/Ni^2+^ 9 and 10.7 times higher than the non-imprinted membrane, respectively. In this way, the imprinted membranes are good for selective silver removal in a solution containing interferent ions such as Cu(II) and Ni(II). 

Another study involving CS was proposed by Wang *et al.* [87], however, in this work, CS was used blended with PVA, in order to obtain a film-forming material, the metal ion-imprinted membrane (IIM) was prepared using silver ions as templates (Ag(I)-IIM). The adsorption capacity of Ag(I)- IIM for Ag(I) is stronger than other ions. Compared with Ag(I)-IIM, non-ion-imprinted membranes (NIIM) for all ions are similar and have a poor adsorption capacity.

### 2.4. Herbicides, Pesticides, Organic Pollutants

Zhu *et al.* [88] prepared a novel thin layer composite MIP membrane selective for monocrotophos (MCP) pesticide by means of *in situ* polymerization of MAA with EGDMA as crosslinker in Nylon-6, introducing specific binding sites into the membrane whilst maintaining its pore structure. Membrane selectivity was evaluated in filtration experiments also using three other organophosphorus pesticides (mevinphos, phosphamidon and omethoate): the composite MIP membrane had low binding affinity for the other pesticides in comparison to the good sorption of the template MCP membrane. 

More recently, Donato *et al.* [89] employed poly-AN and its copolymers with MAA and AAm by a phase inversion technique, using dimethoate as template molecule and testing these membranes against dimethoate and its analog trichlorphon. The membrane obtained with the copolymer P(AN-co-MAA) obtained the best result for binding capacity and selectivity.

Kochkodan *et al.* [90] presented a set of composite membranes imprinted with desmetryn and ibuprofen made with PVDF, both hydrophobic (PVDF_phob) and hydrophilized (PVDF_phil), PSf, polycarbonate and nylon microfiltration membranes as supports for the specifically imprinted materials. 2-acrylamido-2-methyl-1-propane sulfonic acid and MBAA were employed for the preparation of desmetryn-imprinted composite membranes. Dimethylaminoethyl methacrylate and trimethylopropane trimethacrylate were used for the ibuprofen-imprinted membranes. It was observed that the imprinted membranes obtained showed selective binding of structurally similar toxic compounds and selective artificial recognizing elements with high affinity to the template molecules in aqueous solutions.

Prasad *et al.* [91] studied the specific retention of several pesticides: phorate, parathion, atrazine, dichlorovos, ethion, disulfoton, diazinon, 2,4-D, 2,4,5-Twere. For the polymer structure, they employed MAA, EGDMA, di-*n*-octylphthalate, 2-nitrophenyloctyl ether, bis(2-ethylhexyl) sebacate, tris(2-ethylhexyl) phosphate and high molecular mass poly(vinyl chloride).

Having thoroughly studied the effects of pH value, they concluded that the selectivity of the polymer inclusion membranes is remarkable compared to the corresponding non-imprinted ones used in potentiometric sensors. Moreover, the stability, reusability, portability and absence of memory effect mean that the novel phorate sensor device can be readily used in field monitoring.

Vishnuvardhan *et al*. [92] employed a degradation product of Soman, the pinacolyl methylphosphonate, as a template for imprinted polymer materials to create potentiometric sensors. They studied the comparison between three different MIP synthesis methods (bulk, suspension and precipitation) and the selectivity and the sensitivity of the different sensors was bulk > suspension > precipitation.

Pogorelova *et al.* [93] employed molecular recognition sites imprinted in hydrogel films associated with Au–quartz piezoelectric crystals. When the molecule met the recognition site, the hydrogel hydrated and swelled, which could be sensed by microgravimetric quartz crystal microbalance measurements. The imprinted polymer membrane ISFET devices were set up with AAm, sodium methacrylate and MBAA. The triazine herbicides used were atranex (atrazine), prozinex, tyllanex, simanex, ametrex, prometrex, and terbutex.

The imprinted films were immobilized on the gate surface of the ISFETs: the binding of the substrate to the selective site allows electronic transduction. The imprinted films were immobilized on Au–quartz piezoelectric crystals and the binding of the target molecule to the respective imprinted sites caused the polymer film to swell, thereby enabling the microgravimetric analysis of the different herbicides. The complementary electrostatic interactions and H-bonds between the polymerizable monomers and the substrate leads to a successful device for the specific recognition of different triazines.

Haloacetic acids (HAAs) are regulated by the US environmental protection agency (EPA) because of their risks to human health. These acids are monochloroacetic acid (MCAA), monobromoacetic acid (MBAA), dibromoacetic acid (DBAA), dichloroacetic acid (DCAA) and trichloroacetic acid (TCAA). Suedee *et al.* [94] developed sensitive conductimetric sensors for the detection of haloacetic acids (HAAs) in drinking water. They prepared MIPs with 4-vinylpyridine, ethylene glycol dimethacrylate. MIPs were immobilized on the sensor in a PVC membrane (MIP/PVC ratio of 1:2). The recognition selectivity of MIPs for HAAs was TCAA > DCAA > MCAA > DBAA > MBAA > TBAA. 

Xie *et al.* [22] created an electrochemical device for the detection of the organophosphate pesticide chlorpyrifos (CPF), based on a molecular imprinted polymer on gold nanoparticles placed on a glassy carbon electrode (AuNP-gc electrode). CPF molecules assembled on the p-aminothiophenol (ATP) modified AuNP-gc electrode surface were fixed into the imprinted polyaminothiophenol (PATP) membranes and formed superficial imprinted sites. The imprinted PATP-AuNP-gc sensor is about 3.2-fold more sensitive than that of the imprinted PATP-Au sensor, and the sensitivity of the imprinted PATP-AuNP-gc sensor is about two orders of magnitude lower than that of the imprinted PATP-Au sensor. 

In another work, Xie *et al.* [95] presented a study on 2,4-dichlorophenoxy acetic acid (2,4-D), imprinted in polypyrrole polymers (PPy) onto a carbon glass electrode. By performing cyclic voltammetry, it was possible to establish that the device made thus can conspicuously improve the sensitivity and selectivity of 2,4-D analysis, as well as potentially having good repeatability.

**Figure 8:**
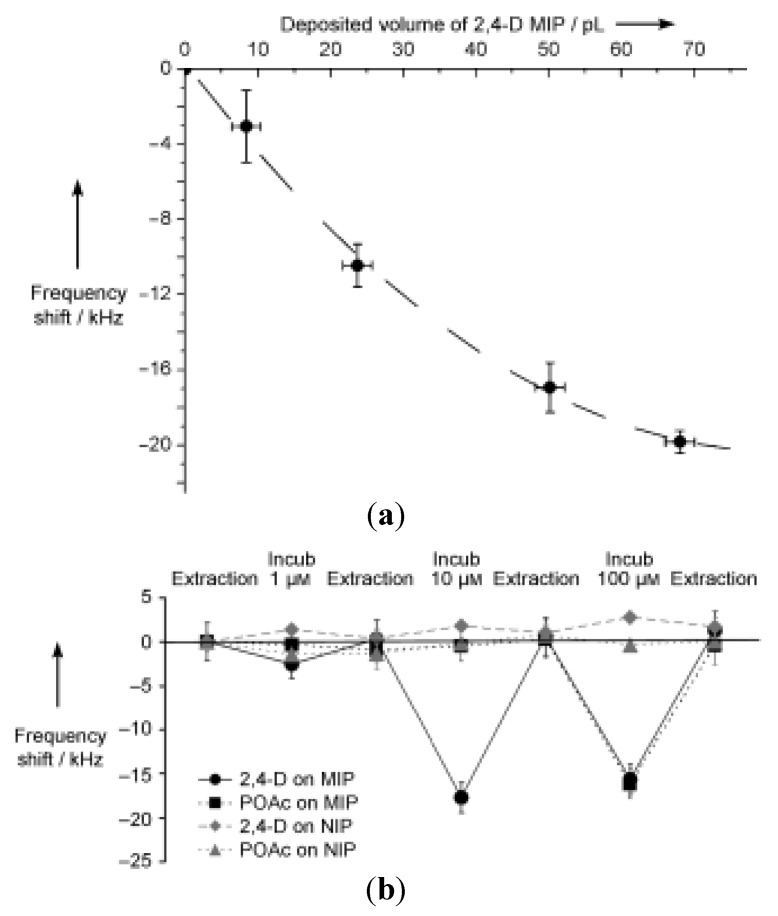
(**a**) Influence of the reduced volume of MIP on the resonance frequency of the array after rebinding of 2,4-D; (**b**) Detection of the rebinding of 2,4-D and POAc at increasing concentrations on a 2,4-D MIP and a NMIP.

The same template molecule 2,4-D was used by Ayela *et al.* [96], to make a combination of silicon microcantilever arrays and MIPs, coupling a resonant electrochemical system (MEMS) to a responsive template layer prepared from 4-VP and trimethylacrylate. The resonance frequency decreased in presence of 2,4-D and subsequently increased when the 2,4-D is not present. The frequency shift depended on the volume of MIP deposited on the micromembrane: increasing the quantity of MIP, the frequency also increased, but tended to plateau values, as is shown in Figure 8a. The selectivity tests were performed with phenoxyacetic acid (POAc) at different concentrations: the array detected only the 2,4-D presence and not the analog POAc (Figure 8b).

The success of photochemical initiation on the synthesis of imprinted membranes has been confirmed in literature. In the study published by Kochkodan et al. [97], PVDF microfiltration membranes were used, both unmodified hydrophobic (PVDF_phob) and hydrophilized (PVDF_phil). The PVDF_phil membrane was previously coated with a thin cross-linked polyacrylate layer over the whole surface of the membrane. The template molecule was the 1,3,5-triazine herbicide desmetryn and it was placed in radical-initiated cross-linking copolymerization with 2-acrylamido-2-methyl-1-propane sulfonic acid (AMPS) as the functional monomer and MBAA as the cross-linker in methanol. The reactions were carried out in two different ways: in variant A the mixture contained the photoinitiator BEE at a concentration of 2.5 mM and UV irradiated after 10 min of soaking; in variant B, the membranes were used coated by soaking in 100 mM solution of BEE in acetone for 5 min and subsequent drying at 40 °C for 15 min. The imprinting effect obtained was significant for thin layers of functional cross-linked copolymers on the surface of PVDF membranes using a coating of a α-scission type photoinitiator. As regards wettability by water, no change was observed for the functionalized PVDF_phil membranes, however the wettability of the PVDF_phob membranes increased with the hydrophilicity of the poly(AMPS-co-MBAA) layer. 

A development of the semi-IPN technique was presented by Sergeyeva *et al.* [98]. In this work the authors prepared a thin and flexible film by adding oligourethaneacrylate to the monomer mixture (MAA/IA/AAm as functional monomers and tri(ethyleneglycol)-dimethacrylate as a crosslinker) for the MIP preparation, maintaining the highly cross-linked polymer feature. The template molecule used was atrazine. The MIP membranes were prepared on the basis of accurate computational modeling. The authors performed the study to verify herbicide recognition properties for the optimization of the composition of atrazine MIP with computational modeling. They identified methacrylic acid to be an optimal functional monomer for atrazine. The computational *in*-*situ*-polymerized MIP membranes obtained showed high adsorption for template molecules (12.5 mg/g_membrane_) and high selectivity also for atrazine analogs.

**Figure 9:**
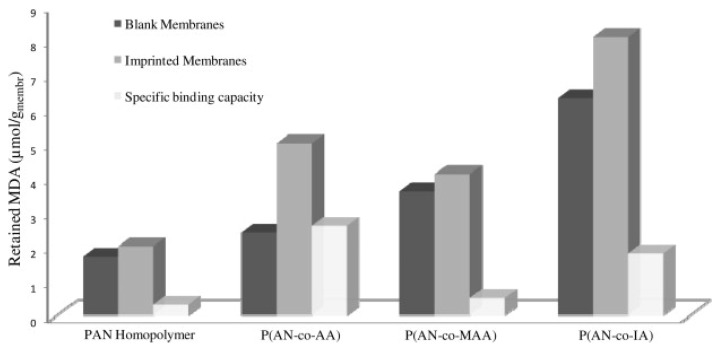
4,4'-methylenedianiline, (MDA) retention and specific binding capacities of the membranes prepared using PAN and the different acrylic copolymers.

Primary amines and some aromatic amines have are considered potentially carcinogens. Del Blanco *et al.* [99] have recently presented a study for the removal of these molecules (in particular 4,4'-methylenedianiline, (MDA)) from organic solvents as impurities or unreacted. They used different AN copolymers such as P(AN-co-IA), P(AN-co-AA) and P(AN-co-MAA), and obtained membranes by phase inversion technique. Binding experiments showed that template membranes are selective for the template molecule (binding capacity: 4.7 μmol/g_membrane_) and distinguished between the template and its analog 4,4'-ethylenedianiline, EDA (binding capacity: 2.9 μmol/g_membrane_). Aniline was also used to test the selectivity of both MIMs and NMIMs and it was seen entirely permeated through blank and imprinted membranes (Figure 9).

**Figure 10:**
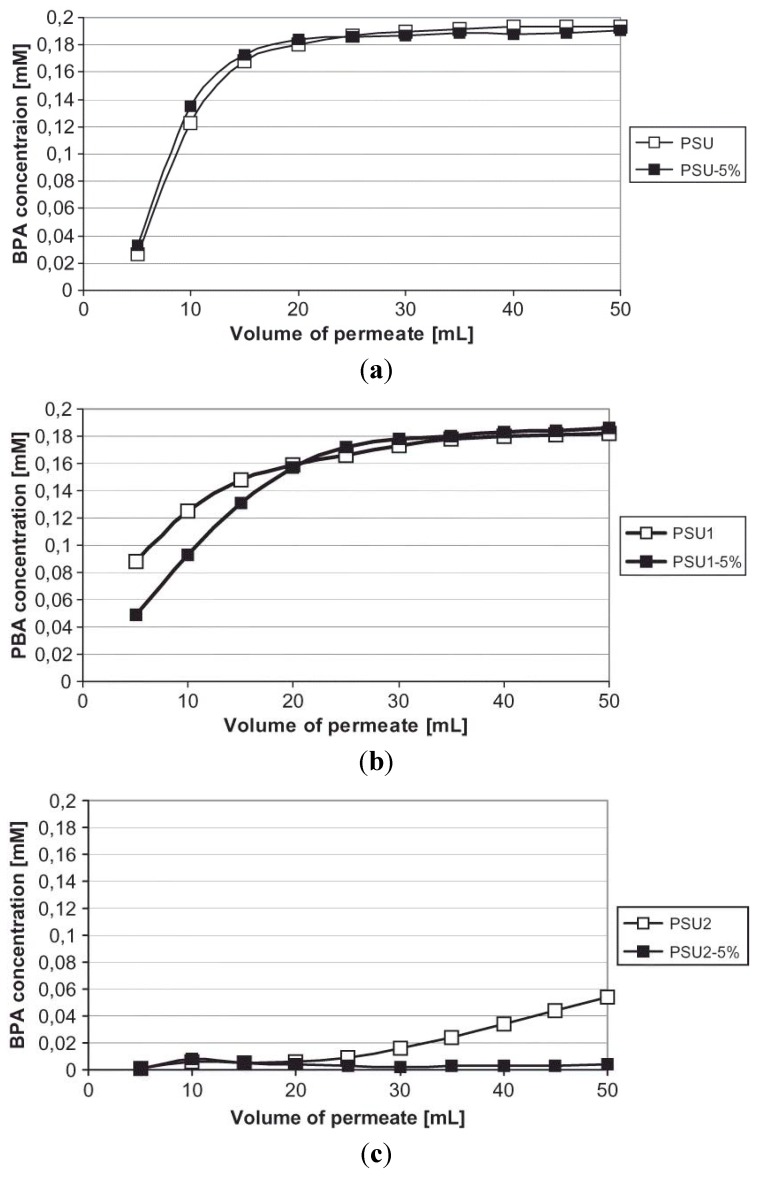
Permeability of PSf membranes with respect to bisphenol A. PSU has degree of sulfonation = 0.00 mol/mer; PSU1 has degree of sulfonation = 0.06 mol/mer; PSU2 has degree of sulfonation = 0.26 mol/mer; 5% is the amount of template (5 wt %).

Bryjak *et al.* [100] reported the preparation and properties of MIMs for the removal of suspect endocrine disruptor bisphenol A by using PSf with different degree of sulfonation (PSU) to prepare MIM by phase inversion technique, templated with bisphenol A. The results have shown that a high content of sulfonic groups (degree of sulfonation = 0.26 mol/mer), made the membranes less permeable to bisphenol A, as it is possible to see in the Figure 10.

Catalytic activity is usually proposed with milled MIPs and not membranes, on account of the powder’s larger surface activity. However, in other cases it is possible to obtain catalytic activity with membranes. Kalim *et al.* [101] proposed two different formats of MIMs to be used for a dehydrofluorination catalysis. The first one was a cellulose filter membrane coated with a polymer generated by MAA, EGDMA and AIBN imprinted with an analog of the chosen reaction (*N*-benzyl-isopropylamine). The second one was prepared by incorporating milled bulk polymers into PVA matrices mixed with glutardialdehyde on a cellulose membrane. Both types of membrane were tested for the catalytic effect on the dehydrofluorination of 4-fluoro-4-(*p*-nitrophenyl)-2-butanone. The coated membranes had no evident catalytic effect, whereas the PVA membranes containing the imprinted polymer particles did show catalytic effects, but these were obtained by allowing the substrate-product mixture to recirculate through the catalytic membrane, because the substrate did not have enough time to interact with the catalyst. 

Sergeyeva *et al.* [102] developed a portable biomimetic sensor device for the specific control of phenol content in water. The synthetic structure reproduced the active site of the enzyme tyrosinase in molecularly-imprinted polymer membranes. Those membranes with a catalytic activity were obtained by co-polymerizing the Cu(II)–catechol–urocanic acid ethyl ester complex with (tri)ethyleneglycoldimethacrylate, adding the elastic component oligourethaneacrylate: this procedure led to the creation of a thin, flexible, and mechanically stable highly cross-linked polymer membrane with catalytic activity. Investigation of the pH-influence demonstrated that pH-dependence peaked at neutral pH values: the oxidation of the catechol is inhibited at pH ≤ 5. In order to examine the selectivity of the new sensor system, catechol analogs (phenol, 4-nitrophenol, 1,2,3-trihydroxybenzol, 2-methoxyphenol, *m*-diphenol, *p*-diphenol, bisphenol A, 1,2-naphthalenediol, and 1,4-naphthalenediol) were added to the electrochemical cell. Unlike conventional biosensor devices made with mushroom tyrosinase that recognize different phenolic compounds, the sensor system developed had high selectivity: it gave catalytic oxidation of *o*-diphenols and no response was observed with their structural analogs (phenol, 4-nitrophenol, and 2-methoxyphenol, *m*-diphenol, *p*-diphenol, bisphenol A, 1,2- naphthalenediol, and 1,4-naphthalenediol). 

## 3. Summary

In order to simplify the comprehension of this work, a summarizing table is proposed, with of template molecules, functional monomers, first authors and year of publication (Table 2).

**Table 2:** A summary of MIMs and templates.

Template	Author	Year	Monomer, Polymer Matrix And Support	Ref.
**Pharmaceutical And Food**				
α-amylase	Silvestri *et al.*	2006	poly(ethylene-co-vinyl alcohol), dextran blends	[43]
	Silvestri *et al.*	2007	poly(ethylene-co-vinyl alcohol)	[44]
α-tocopherol	Faizal *et al.*	2008	α-tocopherol methacrylate, acrylonitrile	[46]
	Faizal *et al.*	2008	α-tocopherol methacrylate, divinylbenzene, polysulfone, cellulose acetate and nylon supports	[47]
	Faizal *et al.*	2009	polysulfone and calix[4]resorcarenes	[48]
(S)-5-benzylhydantoin	Lu *et al.*	2007	poly(styrene-stat-acrylonitrile-stat-vinyl-2,4-diamino-1,3,5-triazine	[15]
BSA	Zhang et al.	2010	acrylamide, multi walled carbon nanotubes	[31]
cholesterol	Sreenivasan *et al.*	1998	hydroxyethyl methacrylate	[28]
cimetidine	Ceolin *et al*.	2009	methacrylic acid, hydroxyethyl methacrylate	[24]
enrofloxacin	Kamel *et al.*	2011	methacrylic acid, 2-vinylpyridine	[62]
folic acid	Donato *et al.*	2010	acrylonitrile, acrylamide	[49]
ibuprofen	Kochkodan *et al.*	2010	dimethylaminoethyl methacrylate, trimethylopropane trimethacrylate	[90]
luteolin	Zhang et al.	2009	3-aminopropyltriethoxysilane	[55]
lysozyme	Chen *et al.*	2010	acrylamide, *N*,*N*-methylene-bis-acrylamide	[58]
methyl orange	Takagishi *et al.*	1972	polyethyleneimine	[3]
naproxen	Ma *et al.*	2010	poly(lactide-co-glycolide) and poly(D,L-lactide)	[50]
naringin	Trotta *et al.*	2002	acrylic acid, acrylonitrile	[56]
	Tasselli *et al.*	2008	acrylonitrile, itaconic acid, acrylic acid, acrylamide	[40]
	Ma *et al.*	2011	chitosan	[16]
N-ethyl-*o*/*p*-toluensulfonamide	Gugliuzza *et al.*	2007	co-poly-(ether/amide)	[9]
	De Luca *et al.*	2009	co-poly-(ether/amide)	[10]
oleanolic acid	Zhang et al.	2011	polyamide-6, poly(styrene-co-maleic acid)	[53]
phosphatidylcoline	Silvestri *et al.*	2007	poly(ethylene-co-vinyl alcohol)	[44]
	Pegoraro *et al.*	2008	poly(ethylene-co-vinyl alcohol)	[45]
propanolol	Yoshimatsu *et al.*	2008	poly(ethylene terephthalate)	[19]
	Jantarat *et al.*	2008	methacrylic acid	[17]
	Yoshimatsu *et al*.	2008	poly(ethylene terephthalate)	[19]
	Renkecz *et al.*	2011	methacrylic acid	[60]
propofol	Petcu *et al.*	2004	4-acetoxystytrene	[59]
puerarin	Quin *et al.*	2011	acrylonitrile, acrylic acid	[52]
rhodamine b	Malaisamy *et al.*	2004	cellulose acetate, polysulfone	[38]
	Ulbricht *et al.*	2005	cellulose acetate, sulfonated polysulfone	[36]
rutin	Zeng *et al.*	2012	acrylamide, 2-vinylpyridine, divinylbenzene	[27]
sulfadiazine	Almeida *et al.*	2011	poly(vinyl chloride)	[64]
sulfamethoxazole	Almeida *et al.*	2011	poly(vinyl chloride)	[64]
tetracycline	Trotta *et al.*	2005	acrylonitrile, acrylic acid	[42]
	Guerreiro *et al.*	2011	methacrylic acid, acrylamide	[63]
theophylline	Kobayashi *et al.*	1995	acrylonitrile, acrylic acid	[32]
	Wang *et al.*	1996	acrylonitrile, acrylic acid	[5]
	Wang *et al.*	1997	*N*,*N*-diethylaminodithiocarbamoylmethylstyrene, acrylic acid	[33]
	Kobayashi *et al.*	1998	acrylonitrile, acrylic acid	[34]
	Hattori *et al.*	2004	methacrylic acid, cellulose	[56]
	Silvestri *et al.*	2006	methyl methacrylate, methacrylic acid	[43]
trimethoprim	Fan *et al.*	2009	methacrylic acid, polysulfone	[29]
	Rebelo *et al.*	2011	methacrylic acid, 2-vinylpyridine	[61]
uric acid	Silvestri *et al.*	2006	acrylonitrile, acrylic acid	[43]
**Aminoacids, Nucleotides And Sugars**				
alanine	Yu *et al.*	2000	acrylic acid	[25]
AMP	Zayats *et al.*	2002	acrylamide, 3-(acrylamido)phenylboronic acid, *N*,*N*-methylene-bis-acrylamide, *N*,*N*,*N*',*N*'-tetramethylethylenediamine	[23]
	Sallacan *et al.*	2002	acrylamide-acrylamidephenylboronic acid copolymer	[79]
5-benzylhydantoin	Lu *et al.*	2007	poly(styrene-stat-acrylonitrile-stat-vinyl-2,4-diamino-1,3,5-triazine)	[15]
CMP	Zayats *et al.*	2002	acrylamide, 3-(acrylamido)phenylboronic acid, *N*,*N*-methylene-bis-acrylamide, *N*,*N*,*N*',*N*'-tetramethylethylenediamine	[23]
	Sallacan *et al.*	2002	acrylamide-acrylamidephenylboronic acid copolymer	[79]
9-ethyladenine	Yoshikawa *et al.*	2001	polystyrene resin, cellulose acetate, polysulfone	[78]
fructose	Sallacan *et al.*	2002	acrylamide, acrylamidephenylboronic acid	[79]
galactose	Sallacan *et al.*	2002	acrylamide, acrylamidephenylboronic acid	[79]
glucose	Sallacan *et al.*	2002	acrylamide, acrylamidephenylboronic acid	[79]
glutamic acid	Yoshikawa *et al.*	1998	carboxylated polysulfone	[67]
	Yoshikawa *et al.*	1998	chloromethylated polystyrene resin, divinylbenzene, dicyclohexylcarbodiimide	[66]
	Yu *et al.*	2000	methacrylic acid	[25]
	Yoshikawa *et al.*	2007	carboxylated polysulfone	[18]
	Yoshikawa *et al.*	2007	myrtenal polysulfone	[74]
	Sueyoshi *et al.*	2010	cellulose acetate	[76]
	Hatanaka *et al.*	2011	polyureas	[75]
	Sueyoshi *et al.*	2012	aldehydic polysulfone	[77]
glutamine	Yoshikawa *et al.*	1998	chloromethylated polystyrene resin, divinylbenzene, dicyclohexylcarbodiimide	[66]
	Reddy *et al.*	2002	nylon-6	[37]
GMP	Zayats *et al.*	2002	acrylamide, 3-(acrylamido)phenylboronic acid, *N*,*N*-methylene-bis-acrylamide, *N*,*N*,*N*',*N*'-tetramethylethylenediamine	[23]
	Sallacan *et al.*	2002	acrylamide-acrylamidephenylboronic acid copolymer	[79]
leucine	Yoshikawa *et al.*	1998	chloromethylated polystyrene resin, divinylbenzene, dicyclohexylcarbodiimide	[66]
lysine	Yoshikawa *et al.*	1998	chloromethylated polystyrene resin, divinylbenzene, dicyclohexylcarbodiimide	[66]
NAD^+^, NADP^+^, NADH, NADPH	Pogorelova *et al.*	2003	acrylamide-acrylamidophenylboronic acid	[80]
phenylalanine	Park *et al.*	2002	acrylic acid	[13]
	Takeda *et al.*	2005	nylon-6, nylon-6,6, terephthalic phenylene polyamide	[70]
	Ul-Haq *et al.*	2008	carboxylated polysulfone	[72]
	Wu *et al.*	2009	sodium alginate, 3-aminopropyltriethoxysilane	[30]
	Ul-Haq *et al.*	2010	acrylonitrile, acrylic acid	[73]
serine	Son *et al.*	2007	polysulfone	[71]
tryptophan	Yoshikawa *et al.*	1997	DLDE derivative	[65]
	Yu *et al.*	2000	acrylic acid, methacrylic acid	[25]
	Itou *et al.*	2008	polystyrene resin	[68]
tyrosine	Dzgoev *et al.*	1999	1,1,1-tris(hydroxymethyl)propane trimethacrylate, methacrylic acid	[69]
	Yu *et al.*	2000	acrylic acid, methacrylic acid	[25]
UMP	Sallacan *et al.*	2002	acrylamide-acrylamidephenylboronic acid copolymer	[79]
uracil	Wang *et al.*	2004	acrylonitrile, methacrylic acid	[39]
	Kobayashi *et al*.	2008	poly(styrene-co-maleic anhydride), poly(styrene-co-maleic acid)	[41]
**Metal Ions**				
[Ni-dipyridyl]^2+^ complex	Wang *et al.*	2008	N-vinyl-2-pyrrolidone	[83]
Ag^+^	Shawky *et al.*	2009	chitosan	[86]
	Wang *et al.*	2009	chitosan, poly(vinylalcohol)	[87]
Cr(NO_3_)_3_·9H_2_O	Chen *et al.*	2010	sodium alginate, poly(vinylalcohol)	[84]
Cu^2+^	Li *et al.*	2007	nitrocellulose, poly(vinyl alcohol)	[81]
	Zhuqing *et al.*	2010	*N*-[3-(trimethoxysilyl) propyl]ethylenediamine	[20]
metallothionein	Cai *et al.*	2008	TiO_2_	[21]
Ni(II)	Vatanpour *et al.*	2011	methacrylic acid	[85]
Zn(II)-(2,2'-bipyridyl)	Zhai *et al.*	2008	4-vinylpyridine	[82]
**Herbicides, Pesticides And Pollutants**				
ametrex	Pogorelova *et al.*	2002	acrylamide, sodium methacrylate, *N*,*N*-methylene-bis-acrylamide	[93]
atranex	Pogorelova *et al.*	2002	acrylamide, sodium methacrylate, *N*,*N*-methylene-bis-acrylamide	[93]
atrazine	Sergeyeva *et al.*	2008	methacrylic acid, itaconic acid, acrylamide	[98]
	Prasad *et al.*	2007	methacrylic acid,	[91]
bisphenol A	Bryjak *et al.*	2011	polysulfone	[100]
chlorpyrifos	Xie *et al.*	2010	polyaminothiophenol	[22]
Cu(II)–catechol–urocanic	Sergeyeva *et al.*	2010	(tri)ethyleneglycoldimethacrylate and oligourethaneacrylate	[102]
2,4-D	Prasad *et al.*	2007	methacrylic acid	[91]
desmetryn	Kochkodan *et al.*	2001	2-acrylamido-2-methyl-1-propane sulfonic acid, *N*,*N*-methylene-bis-acrylamide	[97]
	Kochkodan *et al.*	2010	2-acrylamido-2-methyl-1-propane sulfonic acid, *N*,*N*-methylene-bis-acrylamide	[90]
diazinon	Prasad *et al.*	2007	methacrylic acid,	[91]
dibenzofuran	Kobayashi *et al.*	2002	polysulfone	[35]
2,4-dichlorophenoxy acetic acid	Ayela *et al.*	2007	4-vinylpyridine, trimethylacrylate	[96]
	Xie *et al.*	2010	polypyrrole polymers	[95]
dichlorovos	Prasad *et al.*	2007	methacrylic acid	[91]
dimethoate	Donato *et al.*	2011	acrylonitrile, methacrylic acid, acrylamide	[89]
disulfoton	Prasad *et al.*	2007	methacrylic acid	[91]
ethion	Prasad *et al.*	2007	methacrylic acid	[91]
haloacetic acids	Suedee *et al.*	2004	4-vinylpyridine	[94]
4,4'-methylenedianiline	De Luca *et al.*	2011	acrylonitrile, acrylic acid	[11]
	Del Blanco *et al.*	2012	acrylonitrile, itaconic acid, acrylic acid, methacrylic acid	[99]
monocrotophos	Zhu *et al.*	2006	methacrylic acid, Nylon-6	[88]
*N*-benzyl-isopropylamine	Kalim *et al.*	2005	poly(vinyl alcohol), cellulose acetate	[101]
parathion	Prasad *et al.*	2007	methacrylic acid,	[91]
phorate	Prasad *et al.*	2007	methacrylic acid,	[91]
pinacolyl methylphosphonate	Vishnuvardhan *et al.*	2007	methylmethacrylic acid	[92]
prometrex	Pogorelova *et al.*	2002	acrylamide, sodium methacrylate, *N*,*N*-methylene-bis-acrylamide	[93]
prozinex	Pogorelova *et al.*	2002	acrylamide, sodium methacrylate, *N*,*N*-methylene-bis-acrylamide	[93]
simanex	Pogorelova *et al.*	2002	acrylamide, sodium methacrylate, *N*,*N*-methylene-bis-acrylamide	[93]
terbutex	Pogorelova *et al.*	2002	acrylamide, sodium methacrylate, *N*,*N*-methylene-bis-acrylamide	[93]
1,3,5-triazine	Kochkodan *et al.*	2001	2-acrylamido-2-methyl-1-propane sulfonic acid, *N*,*N*-methylene-bis-acrylamide	[97]
2,4,6-trichlorophenol	Feng *et al.*	2008	4-vinylpyridine, methyacrylic acid	[26]
tyllanex	Pogorelova *et al.*	2002	acrylamide, sodium methacrylate, *N*,*N*-methylene-bis-acrylamide	[93]
2,4,5-twere	Prasad *et al.*	2007	methacrylic acid	[91]

## 4. Conclusions

Molecular imprinting membranes appears to be one of the most promising separation and recognition technologies in terms of discrimination and versatility.

Moreover, the great number of polymers and techniques currently available allow the preparation of a large set of membranes with different functional groups and recognition sites, thus extending the application of such technology as proven by the number of papers reported by the literature in the past year.

## Nomenclature

AA: acrylic acid
AAm: acrylamide
AIBN: 2,2'-azobisisobutyronitrile
AMP: adenosine 5'-monophosphate
AN: acrylonitrile
APTES: 3-aminopropyltriethoxysilane
α-TMA: α-tocopherol methacrylate
Boc-: *N*-α-*tert*-butoxycarbonyl protection
Bzl: benzyl protection
CA: cellulose acetate
CMP: cytosine 5'-monophosphate
CS: chitosan
DLDE: H-Asp(OcHex)-Leu-Asp(OcHex)-Glu(OBzl)-CH_2_-
DMF: *N*,*N*'-dimethylformamide
DMSO: dimethylsulfoxide
EGDMA: ethylene glycol dimethacrylate
ESD: electrospray deposition
EVA: poly(ethylene-co-vinyl alcohol)
Gln: glutamine
Glu: glutamic acid
Gly: glycine
GMP: guanosine 5'-monophosphate
IA: itaconic acid
iniferter: initiator-transfer agent-terminator
IPN: interpenetrating polymer
ISFET: ion sensitive field-effect transistor
Leu: leucine
Lys: lysine
MAA: methacrylic acid
MBAA: *N*,*N*-methylene-bis-acrylamide
MIM: molecularly imprinted membrane
MIP: molecularly imprinted polymer
MIPCM: molecular imprinted polymer composite membrane
MMA: methylmethacrylic acid
NAD^+^/NADH: nicotinamide adenine dinucleotide/reduced nicotinamide adenine dinucleotide
NADP^+^/NADPH: nicotinamide adenine dinucleotide phosphate/reduced nicotinamide adenine dinucleotide phosphate
NG: naringin
NHD: neohesperidin
NMIM: non molecularly imprinted membrane
NMIP: non molecularly imprinted particle
NMP: *N*,*N*-methylpyrrolidone
Ny: nylon
P(AN-co-AA): poly(acrylonitrile-co-acrylic acid)
P(AN-co-AAm): poly(acrylonitrile-co-acrylamide)
P(AN-co-DTCS): poly(acrylonitrile-co-*N*,*N*'-diethylaminodithiocarbamoylmethylstyrene)
P(AN-co-IA): poly(acrylonitrile-co-itaconic acid)
P(AN-co-MAA): poly(acrylonitrile-co-methacrylic acid)
PA6: polyamide-6
PAN: polyacrylonitrile
PC: phosphatidylcoline
PEG: polyethylene glycol
PET: poly(ethylene terephthalate)
Phe: phenylalanine
PP: poly(propylene)
PSf: polysulfone
PSMA: poly(styrene-co-maleic acid)
PTFE: polytetrafluoroethylene
PU: polyurethane
PVA: poly(vinyl alcohol)
PVC: poly(vinyl chloride)
PVDF: polyvinylidene fluoride (PVDF_phil**:** hydrophilized polyvinylidene fluoride; PVDF_phob: unmodified hydrophobic polyvinylidene fluoride)
QCM: quartz crystal microbalance
RT: rutin
SA: sodium alginate
ScCO_2_: supercritical carbon dioxide
Ser: serine
TCH: tetracycline hydrochloride
TEOS: tetraethoxysilane
THF: tetrahydrofuran
TMP: trimethoprim
Trp: tryptophan
Tyr: tyrosine
UMP: uridine 5'-monophosphate
VP: vinylpyridine
